# Supplementary material for: Asynchronous magnetic resonance elastography: Shear wave speed reconstruction using noise correlation of incoherent waves
Source: Magn Reson Med. 2022 Oct 27;89(3):990–1001. doi: 10.1002/mrm.29502 (PMC9792433; doi:10.1002/mrm.29502)
Supplement: Supplementary file 1 — DATA S1 MATLAB code used to generate the numerical simulations of Figure 1 [file MRM-89-990-s002.zip › k-Wave/helpfiles/loadImage.html]

loadImage :: Functions (k-Wave)


# loadImage

Load an image file.

## Syntax

```
im = loadImage()
im = loadImage(filename)
```

## Description

`loadImage` loads an external image file using `imread` and returns a two-dimensional image matrix scaled between 0 and 1. If a colour image is loaded, the colour channels are summed before scaling. If no input is given for `filename`, a selection dialog is invoked.

## Optional Inputs

|  |  |
| --- | --- |
| `filename` | filename (and pathname if not in the same directory) of the image to load |

## Outputs

|  |  |
| --- | --- |
| `im` | scaled image matrix |

## Examples

- Loading External Image Maps
- 2D Time Reversal For A Circular Sensor
- Optimising k-Wave Performance

## See Also

`imread`, `resize`
